# *B.2.16* is a non-lethal modifier of the *Dark^82^* mosaic eye phenotype in *Drosophila melanogaster*

**DOI:** 10.17912/micropub.biology.000359

**Published:** 2021-01-18

**Authors:** Alysia D Vrailas-Mortimer, Nitish Aggarwal, Neha N Ahmed, Ian M Alberts, Masa Alhawasli, Ibrahim A Aljerdi, Brooke M Allen, Ahmad M Alnajar, Michael A Anderson, Ryan Armstong, Chace C Avery, Eric J Avila, Tyra N Baker, Soolmaz Basardeh, Natasha A Bates, Farrah N Beidas, Ashton C Bosler, Deja M Brewer, Ryan S Buenaventura, Natalie JL Burrell, Alyn P Cabrera-Lopez, Amairani B Cervantes-Gonzalez, Raymond P Cezar, Joselyn Coronel, Corinne Croslyn, Konnor R Damery, Lucero Diaz-Alavez, Nupur P Dixit, Dilcia L Duarte, Amanda R Emke, Katherine English, Audrey A Eshun, Samuel R Esterly, Arnold J Estrada, Mark Feng, Meghan M Freund, Niko Garcia, Charandeep S Ghotra, Haddia Ghyasi, Christa SA Hale, Luci Hulsman, Lindsey Jamerson, Austin K Jones, Madeline Kuczynski, Takailah N Lacey-Kennedy, Mindy J Lee, Tala Mahjoub, Molly C Mersinger, Alyssa D Muckerheide, David W Myers, Karen Nielsen, Paul J Nosowicz, Jacinda A Nunez, Amy C Ortiz, Tulsi T Patel, Natalie N Perry, W Storm A Poser, Deisy M Puga, Cathrine Quam, Paulina Quintana-Lopez, Piper Rennerfeldt, Nicholas M Reyes, Ian G Rines, Cally Roberts, Daniel B Robinson, Kalina M Rossa, Gloria J Ruhlmann, Jeremy Schmidt, John R Sherwood, Dimyana H Shonoda, Hannah Soellner, Juan C Torrez, Mayukah Velide, Zachary Weinzapfel, Alisa C Ward, Kayla L Bieser, Julie A Merkle, Joyce C Stamm, Richard L Tillett, Jacob D Kagey

**Affiliations:** 1 Illinois State University, Normal, IL USA; 2 University of Detroit Mercy, Detroit, MI USA; 3 University of Evansville, Evansville, IN USA; 4 Nevada State College, Henderson, NV USA; 5 University of Nevada Las Vegas, Las Vegas, NV USA

## Abstract

Genetic screens have been used to identify genes involved in the regulation of different biological processes. We identified growth mutants in a Flp/FRT screen using the *Drosophila melanogaster* eye to identify conditional regulators of cell growth and cell division. One mutant identified from this screen, *B.2.16*, was mapped and characterized by researchers in undergraduate genetics labs as part of the Fly-CURE. We find that *B.2.16* is a non-lethal genetic modifier of the *Dark^82^* mosaic eye phenotype.

**Figure 1. The B.2.16, Dark82 mosaic eye results in an increase in the ratio of mutant pigmented (w+mC) tissue compared to wild type unpigmented tissue:**
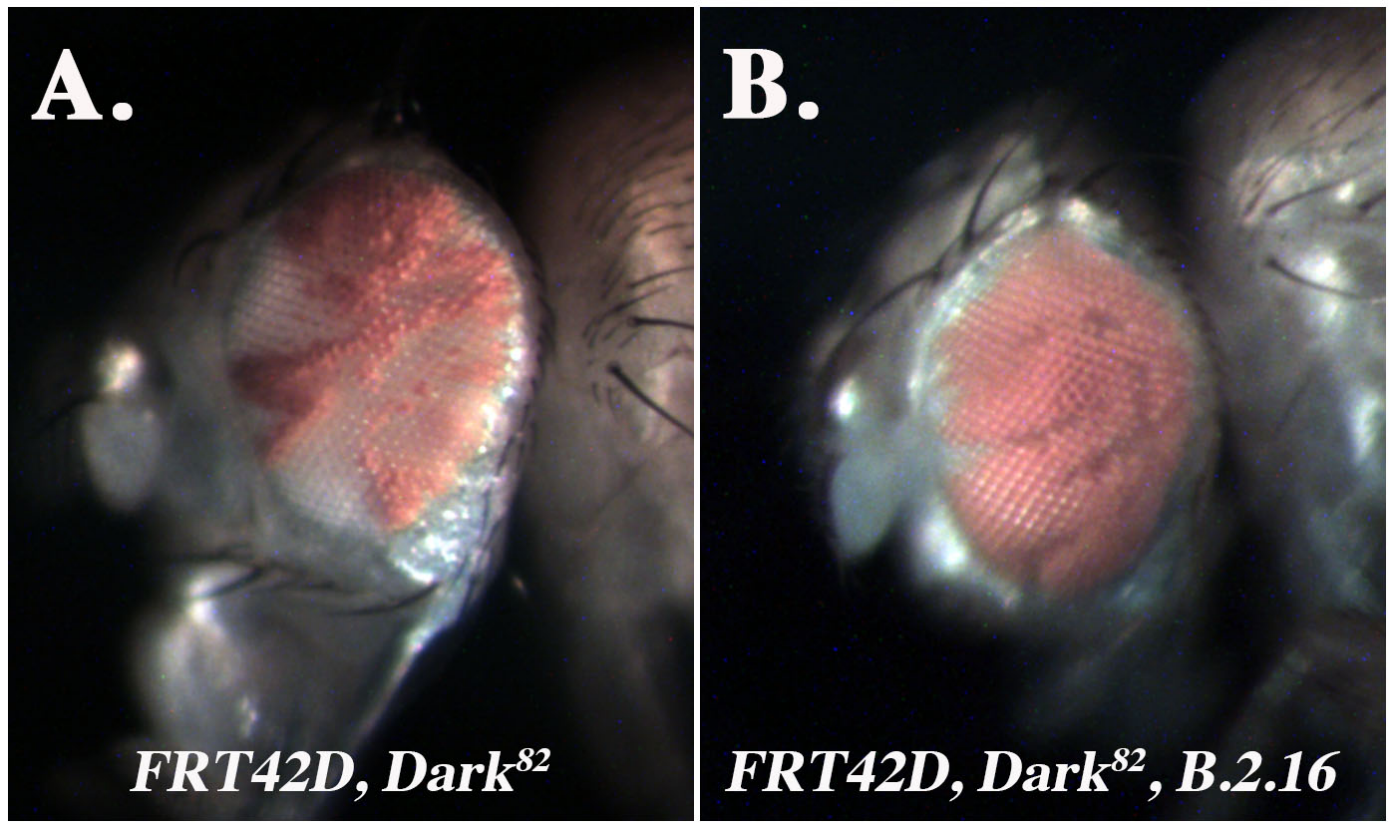
A. *Dark^82^* mosaic eye (control), mutant tissue is pigmented. B. *Dark^82^, B.2.16* mosaic eye, mutant tissue is pigmented.

## Description

Table 1: Complementation tests conducted with mutant B.2.16

**Table:** 

**2R deficiency kit overlapping *Dark***			
*Deficiencies tested within Dark deficiency region*			
**Deficiency**	**BDSC Stock #**	**Region**	**Complementation test with *B.2.16***
*Df(2R)BSC382*	24406	2R: 16,776,164..16,901,625	Complement
*Df(2R)CG15614^attP^*	84470	2R: 16,945,107..16,947,153	Complement
*Df(2R)ED1*	6916	2R: 17,026,727..17,097,322	Complement
*Df(2R)BSC433*	24937	2R: 17,062,915..17,097,315	Complement
*Single genes tested within Dark deficiency region*			
**Gene**	**BDSC Stock #**	**Allele**	**Complementation test with *B.2.16***
*Ehbp1*	10931	*Ehbp1^k09837^*	Complement
*CG8963*	12432	*CG8963^BG00665^*	Complement
*Pkc53E*	20790	*Pkc53E^EY14093^*	Complement
*mute*	22655	*mute^EY22147^*	Complement
*PIG-V*	32781	*PIG-V^MI01787^*	Complement
*AsnRS-m*	10671	*AsnRS-m^k07408^*	Complement
*CG6805*	78974	*CG6805^CR00657-TG4.0^*	Complement
*CG30460*	59338	*CG30460^MI134331^*	Complement
**Genes tested outside of 2R deficiency kit**			
**Gene**	**BDSC Stock #**	**Allele**	**Complementation test with *B.2.16***
*l(2)41ab*	62050	*l(2)41Ab^KV00249^*	Complement
*CG40191*	62029	*CG40191^KV00004^*	Complement

In order to identify genes that contribute to the genetic regulation of *Drosophila* eye development and the regulation of tissue growth, an ethyl methanesulfonate (EMS) mutagenesis screen was performed utilizing the FLP/FRT system, which generates genetically mosaic clones in a tissue specific manner. The screen was performed starting with a copy of chromosome 2R carrying an allele of *Death-associated APAF1-Related Killer* (*Dark),* *Dark^82^*, which was generated by imprecise P-element excision and therefore retains eye pigmentation due to the presence of an insertion encoding *mini-white* (Akdemir *et al.* 2006). When homozygous, the *Dark^82^* allele blocks cell death and allows for the detection of conditional regulators of eye development and tissue growth that might otherwise induce apoptosis (Kagey *et al.* 2012). Flies that bear a *FRT42D, Dark^82^* chromosome (2R) were mutagenized with EMS and then crossed to *ey-Flp; FRT42D* flies. The mosaic eyes of resulting offspring were analyzed for phenotypes associated with overall head and eye size, ratio of mutant to wild-type tissue (red over white), or developmental patterning defects (Kagey *et al.* 2012). One mutant from this screen, *B.2.16*, was selected for study here. The control mosaic eye, *Dark^82^*, has an average ratio of 50-60% mutant (pigmented) tissue (**Figure 1A**). In comparison the *B.2.16, Dark^82^* mosaic eye ranges from 75-80% mutant tissue (*w^+mC^)* demonstrating a consistent increase in the amount of mutant tissue from the *Dark^82^* mosaic ratio *(***Figure 1B**). To understand the mechanism driving this increase in mutant tissue overrepresentation, *B.2.16* was mapped via complementation mapping by undergraduate researchers who were part of the Fly-CURE consortium. This type of mapping has been successful for previous mutants from this screen such as *Egfr, Ptc,* and *Shn* (Bieser *et al.* 2019, Kagey *et al.* 2012, Stamm *et al.* 2019)

The genetic mapping of *B.2.16* on chromosome 2R was completed by four independent groups of undergraduate researchers at Illinois State University, Nevada State College, the University of Evansville, and the University of Detroit Mercy as a part of the Fly-CURE consortium (Bieser *et al.* 2018, Bieser *et al.* 2019, Stamm *et al.* 2019). Complementation tests were conducted using the 87 deficiency stocks from the Bloomington Stock Center 2R Deficiency Kit that are distal to the *FRT42D* location (Cook *et al.* 2012). With the hypothesis that the *B.2.16* mutation is homozygous lethal, we mapped homozygous lethality by performing complementation tests using the 87 deficiency stocks. Virgin *FRT42D, B.2.16, Dark^82^/CyO* females were crossed to males from each of these deficiency stocks and the F1 progeny were scored for complementation. None of the deficiency stocks failed to complement the *B.2.16* mutation. This finding suggests three possible hypotheses: 1) The *B.2.16* mutation is linked with the *Dark^82^* mutation and therefore unable to be mapped via complementation due to the homozygous lethal nature of the *Dark^82^* allele. Two deficiencies from the deficiency kit (*Df(2R)ED2747* and *Df(2R)BSC331*) fail to complement *Dark^82^*; if the *B.2.16* mutation lies within this region of 2R:16,869,330..17,097,303, then a failure to complement for the *Dark^82^* allele would be indistinguishable from a failure to complement for the *B.2.16* allele (Gramates *et al.* 2017). 2) The *B.2.16* mutation lies in a region that is not covered by a deficiency stock. There are 46 predicted genes on chromosome 2R that are not covered by the deficiency kit (https://bdsc.indiana.edu/stocks/df/dfkit-info.html, Cook *et al.* 2012). If the mutation exists in one of these genes, it cannot be mapped using the deficiency kit. 3) Lastly, the *B.2.16* mutation is not a homozygous lethal mutation and therefore cannot be mapped via complementation tests.

We conducted additional experiments to investigate these hypotheses. To test the first hypothesis that the *B.2.16* mutation is tightly linked to the lethal *Dark^82^*,we set up complementation tests with smaller deficiency stocks and all available individual gene lethal alleles within the *Dark^82^* region (see Table 1 for a list of stocks tested). We found that *B.2.16* complemented all of these crosses within the *Dark^82^* region, suggesting that *B.2.16* is not a lethal mutation tightly linked to *Dark*. To test the second hypothesis that *B.2.16* lies in a region of 2R that is not covered by the deficiency kit, we investigated the 46 predicted genes that are not covered by the Df kit. Of these predicted genes, 33 are considered either pseudo-genes or ‘gene model not supported’ on FlyBase and therefore are unlikely candidates (Thurmond *et al.* 2019). For the remaining 13 genes, there were two available lethal alleles, which were tested and shown to complement with *B.2.16* (Table 1). Since our testing for complementation is limited to available lethal alleles and deficiencies, we also investigated genome-wide sequencing (GWS) data for *B.2.16* to look for changes in any gene not directly tested via complementation. We found no mutations within these untested genes (either the *Dark* overlap region or in areas not covered by the Df kit) that would truncate or alter protein function, per SNP effect predictions by SnpEff software (Cingolani *et al.* 2012).

Given that the *B.2.16* mutation complemented all stocks tested across 2R (Df Kit, additional Df stocks, and single allele stocks) and that GWS provides no clear candidate mutations within the genes not tested by complementation, we conclude that the *B.2.16* mutation is a non-lethal mutation that modifies the *Dark^82^* mosaic eye phenotype resulting in an increased ratio of mutant tissue to wild-type tissue.

## Reagents

*FRT42D Dark^82^/CyO* (Akdemir *et al..*, 2006)*FRT42D Dark^82^, B.2.16 /CyO* (this manuscript)*Ey-Flp; FRT42D* (BDSC 8211) Bloomington Drosophila Stock Center 2R Deficiency Kit (Cook *et al.*,2012)

Additional Bloomington Stocks (See Table 1 for complete list of stock numbers)
